# Schizophrenia and Influenza at the Centenary of the 1918-1919 Spanish Influenza Pandemic: Mechanisms of Psychosis Risk

**DOI:** 10.3389/fpsyt.2020.00072

**Published:** 2020-02-26

**Authors:** Adrianna P. Kępińska, Conrad O. Iyegbe, Anthony C. Vernon, Robert Yolken, Robin M. Murray, Thomas A. Pollak

**Affiliations:** ^1^ Department of Psychosis Studies, Institute of Psychiatry, Psychology and Neuroscience, King's College London, London, United Kingdom; ^2^ Department of Basic and Clinical Neuroscience, Institute of Psychiatry, Psychology and Neuroscience, King’s College London, London, United Kingdom; ^3^ MRC Centre for Neurodevelopmental Disorders, King’s College London, London, United Kingdom; ^4^ Stanley Laboratory of Developmental Neurovirology, Johns Hopkins Medical Center, Baltimore, MD, United States

**Keywords:** infection, epidemiology, autoimmunity, neurodevelopment, maternal immune activation (MIA), influenza, schizophrenia

## Abstract

Associations between influenza infection and psychosis have been reported since the eighteenth century, with acute “psychoses of influenza” documented during multiple pandemics. In the late 20^th^ century, reports of a season-of-birth effect in schizophrenia were supported by large-scale ecological and sero-epidemiological studies suggesting that maternal influenza infection increases the risk of psychosis in offspring. We examine the evidence for the association between influenza infection and schizophrenia risk, before reviewing possible mechanisms *via* which this risk may be conferred. Maternal immune activation models implicate placental dysfunction, disruption of cytokine networks, and subsequent microglial activation as potentially important pathogenic processes. More recent neuroimmunological advances focusing on neuronal autoimmunity following infection provide the basis for a model of infection-induced psychosis, potentially implicating autoimmunity to schizophrenia-relevant protein targets including the N-methyl-D-aspartate receptor. Finally, we outline areas for future research and relevant experimental approaches and consider whether the current evidence provides a basis for the rational development of strategies to prevent schizophrenia.

## Introduction: What Is the Evidence for an Association Between Influenza and Schizophrenia?

Schizophrenia risk is associated with a variety of environmental and genetic factors (1), including those associated with immunity and inflammation (2). Genome-wide association studies (GWAS) implicate loci at the major histocompatibility complex (MHC) which encodes multiple genes involved in immunity such as the *human leukocyte antigen* (*HLA*) genes (3–6) and complement component 4 (C4) (7), among others, and enhancers related to B-lymphocyte lineages (CD19 and CD20 lines) involved in acquired/adaptive immunity (8). Overall there is strong evidence supporting the involvement of specific immune variants in schizophrenia risk (7), some evidence of convergence across genomics, transcriptomic, and methylomic processes (9), but conflicting evidence for both (i) enrichment of specific immune cell types or pathways (10) and (ii) for genetic overlap between SZ and specific immune diseases (11, 12).

Substantial epidemiological evidence exists suggesting that maternal, perinatal, childhood, and adult infection may all increase the risk of schizophrenia diagnosis (13–19). While many organisms and infection types have been implicated in schizophrenia risk, the influenza virus has special status: not only is maternal influenza infection the most well-replicated infective risk factor for schizophrenia, but the history of schizophrenia research has been shaped at crucial points by observations concerning the apparent, sometimes surprising, role of influenza as an exposure. This review aims to present the current state of knowledge on mechanisms by which influenza infection may confer schizophrenia risk, along with the implications of this understanding for future research, prevention, and treatment.

Before the focus of this review moves to schizophrenia and related psychotic disorders, it should be noted that some of the associations that will be discussed are now thought not to be specific to schizophrenia risk. The late winter/spring season of birth effect has also been reported in bipolar disorder (BD) (20), but the evidence for a link between BD and influenza is somewhat mixed (21) and addressed in limited studies (22). Influenza (including serologically documented infection) has been reported as a risk factor for BD with psychotic features but not nonpsychotic BD [reviewed in (22–24)]. Furthermore, some evidence suggests an association between maternal infection and autism spectrum disorders (ASD) [reviewed in (25)]. A substantial body of work from Scandinavian (largely Danish) health register studies supports the notion that clinically diagnosed maternal, childhood, or adulthood infection is a pluripotent risk factor for the subsequent development of psychiatric disorder, with effects observed across diagnostic boundaries (13–16, 26, 27). Therefore, while the focus of this review is on schizophrenia and psychosis, the potential transdiagnostic relevance of some of the mechanisms reviewed here should not be ignored.

Currently, influenza is regarded predominantly as a respiratory illness, but before the last century a far broader conceptualisation existed. As early as a 1732 epidemic, clinicians made note of the nervous sequelae of infection, with manifestations including neurasthenia, melancholy, hysteria, mental prostration, and insanity (28). According to the historian of medicine Mark Honigsbaum: “in the mid-1890s British medical journals were full of tales of Victorian professionals driven to the brink of madness and beyond by the nervous sequelae of influenza… for some 30 years, from the first epidemics of Russian influenza in the 1890s through to the ‘Spanish’ influenza of 1918–19, the ‘psychoses of influenza' were a widely recognised psychiatric phenomenon” (29). In 1919, Karl Menninger published a now-classic paper reporting the characteristics of 100 patients with mental disturbances associated with influenza infection admitted in a 3-month period to the Boston Psychopathic Hospital. Of 80 on whom full data were available, 16 were diagnosed with delirium, 25 with “dementia praecox,” 23 with “other psychoses,” and 16 were unclassified (30). Interestingly, two-thirds of the “dementia praecox” patients were reported to have fully recovered at 5-year follow up (31). A further historically important strand of evidence came from von Economo's (32) research on encephalitis lethargica (EL), a still poorly understood inflammatory CNS condition featuring psychotic and catatonic symptoms, which was broadly contemporaneous with and potentially aetiologically related to the 1918-1919 Spanish influenza pandemic. **Table 1** provides an overview of historical influenza pandemics that have been linked to the occurrence of psychosis.

**Table 1:** Influenza pandemics and their relationships to psychosis.

Name of influenza pandemic	Dates	Influenza strain involved	Relationship to psychosis	References
1889–1892 influenza pandemic (Russian influenza)	1889–1892	H2N2	psychosis, suicidal thoughts, paranoia following infection	(29, 33, 34)
1918 Spanish influenza pandemic	1918–1920	H1N1	delirium, dementia praecox, acute psychosis (35); encephalitis lethargica (32) following infection	(31, 32, 35)
Asian influenza pandemic	1957–1958	H2N2	acute psychotic manifestations: anxiety, confusion, restlessness, paranoia, abnormal electroencephalography 2-10 days after influenza onset, (36); excess of female births with an increased schizophrenia risk five months after the onset of the 1957 epidemic (37); however, no significant excess of schizophrenia cases in births in the 1959 epidemic (37);	(33, 36–43)
2009 influenza pandemic (swine flu)	2009–2010	H1N1	encephalitis, psychosis, including depressive-type psychosis and repetitive transient psychosis in children following infection	(44, 45)

While suggestive, these reports do not provide evidence of a causal link between influenza infection and psychotic disorders. Renewed interest in the second half of the 20^th^ century shifted focus towards maternal infection, following consistent findings of an increased risk for schizophrenia in late winter/spring season births (20, 46), raising the possibility of winter-borne infection as a plausible mechanism. Beginning with Mednick et al.'s 1988 study of a Finnish population exposed to the 1957 influenza A2 pandemic, epidemiological studies in the 1980s–1990s, of an ecological nature, described increased risk for schizophrenia in children who were *in utero* during an influenza epidemic (37–41). These studies are comprehensively reviewed in (47). Frequently, rates were highest for second trimester exposures, although the first trimester appeared also to be a period of increased risk. Some subsequent studies however—often with more accurate case ascertainment and larger samples—were not able to replicate these initial findings [e.g. (48, 49)]. While estimates of risk varied greatly and heterogeneity in methodology somewhat limits generalisability, a 2010 review calculated that maternal influenza exposure increased schizophrenia risk with an odds ratio of 3.0 and a population attributable proportion of 14% (47).

Partly because of the manifold methodological problems involved in imputing precisely who was exposed to influenza, these ecological studies were followed by so-called “sero-epidemiological” studies, in which infection was verified using archived biological specimens: in one such early study first trimester maternal exposure was associated with a sevenfold increase in offspring schizophrenia risk, with threefold increase in risk associated with early-to-mid gestation exposure (50).

Other studies explored whether other viral and bacterial infections are associated with differential schizophrenia risk. A meta-analysis found that childhood viral infection was associated with a nearly twofold increased risk of adult nonaffective psychosis and that of all childhood infections, viral infections in particular, were associated with a nearly twofold increased risk of adult schizophrenia (18). However, bacterial infections were not associated with risk for psychosis, suggesting that risk may be specific for childhood viral infections.

Some controversy persists as to whether the evidence for maternal influenza as a schizophrenia risk factor is sufficient. A recent review of studies of schizophrenia risk in relation to the 1957 influenza pandemic criticised the serological studies for using strain-specific antibody titres that were too low to be specific for recent infection and so were insufficient as proxy measures of recent infection; furthermore, a pooled meta-analysis of eight ecological studies and one serological study found no overall increased risk of schizophrenia in children of influenza-exposed mothers (51). This review was in turn criticised as inappropriate given the heterogeneity of methods used in the pooled studies (52); furthermore it appeared to omit some relevant serological data [e.g. (39, 40)] and as it focuses on the 1957 pandemic only, it does not include studies on other strains of influenza infection and psychosis.

Complicating interpretation of ecological/epidemiological and serological studies is the fact that obstetric complications are more likely following influenza or influenza-like illness, and that obstetric complications are an independent risk factor for the subsequent development of psychotic disorders and/or symptoms (53–55).

### Reconciling Maternal Infection With Influenza With the Neurodevelopmental Hypothesis of Schizophrenia

The late 1980s and 1990s saw the emergence of neurodevelopmental theories offering mechanistic accounts of how schizophrenia develops. The neurodevelopmental hypothesis (56, 57) posits that schizophrenia results from a pathological disruption of normal brain development which commences many years before schizophrenia onset (58). Infection and other insults could disrupt developmental processes such as cell proliferation, cell migration, arborisation, and myelination (59) with resulting brain structural alterations [e.g. ventricular enlargement, grey matter reductions, and white matter disruption; (60)]; activation of pathologically developed brain systems in adolescence or young adulthood then manifests in schizophrenia symptoms (59).

Amongst other criticisms, the theory fails to account for later-onset schizophrenia [45 years or older; (61)] and postadolescence changes (62). Extended neurodevelopmental models posited further “hits,” e.g., genetic and environmental factors first predisposing to schizophrenia prenatally and then later in life [“three-hit” model, (63); multiple hit theory, (64)]. Infection is a possible “hit”; for instance, human endogenous retrovirus infections, activated by viruses including influenza, were suggested as late “hits” (64). This theory is consistent with evidence that maternal infection contributes to later increased offspring risk for childhood infections, which in turn contribute to schizophrenia development (65).

There is no clear evidence that genetic liability to schizophrenia increases the likelihood of influenza infection or predisposes to a disrupted immune response to influenza, or that influenza genetic risk loci are implicated in schizophrenia. In terms of genetic risk for influenza infection, while significant genetic effects accounting for the antibody level in influenza A and B (66, 67) have been reported with *h* ^2^ (heritability) range of 0.20–0.27 and *c* ^2^ (shared environnment) = 0.19 for influenza A and B (66), results for discrete serostatus (seropositive/seronegative) were significant for influenza B only. However, another GWAS of IgG response to viruses identified HLA class II residues as causal variants and found an overlap between variants affecting the humoral response to influenza A and variants linked to influenza-related autoimmune disorders including narcolepsy (68). Neither of these studies nor any others to date have directly addressed the issue of overlap between genetic risk for schizophrenia and specific risk for influenza infection, although this has been explored for other pathogens (69–71). A UK population-based cohort study of 7,921 mothers found no association between schizophrenia polygenic risk score (PRS) and perinatal infection (using a single “any infection” category) (72). Similarly, a case-control study by Benros et al. explored an association between schizophrenia PRS and a history of hospital contacts for viral infections, including influenza infection: PRS for schizophrenia did not account for the association between hospitalisation for infection and subsequent schizophrenia risk, indicating that schizophrenia risk does not increase proneness to such severe infections (73).

## The Influenza Virus and Potential Pathological Mechanisms Underlying the Association Between Schizophrenia and Influenza Infection

### Influenza: Structure and Pathophysiology

**Figure 1:**
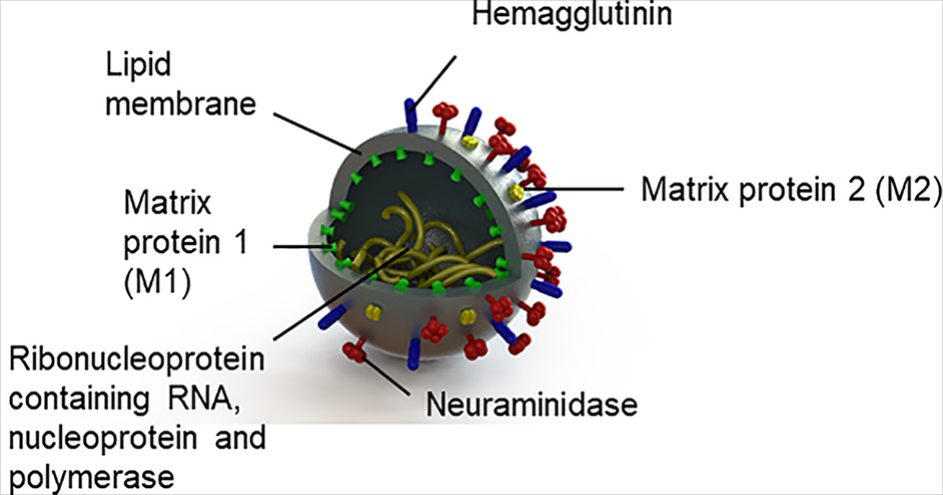
Structure of the influenza virus.

The influenza virus is an enveloped RNA virus from the family *Orthomyxoviridae*, with three genera, influenza A, B, and C (74). Given that influenza type A is responsible for pandemics (75) historically linked to schizophrenia and psychotic symptoms (see **Table 1**), we will focus on this alone. Influenza A viruses are classified into subtypes based on the antigenic properties of their envelope glycoproteins (see **Figure 1**), hemagglutinin, and neuraminidase. The viral envelope is a lipid membrane derived from plasma membrane of an infected host cell. Influenza strain targets also differ. Notably, the H5N1 virus and other avian-derived strains are neurotropic while H1N1 is thought not to be (76–78).

For infection to be successful, hemagglutinin binds the influenza virus to its receptors, sialyloligosaccharides, on the host cell surface. The viral envelope and the host cell membrane fuse giving the viral RNA access to the host cell (74, 79). Neuraminidase facilitates virus release (74, 75). Following the production of viral particles in the nucleus of the host cell, the host cell lyses and dies (75). Protective immune responses from the cell occur; the viral hemagglutinin, neuraminidase, and matrix 2 (M2) proteins are targeted by antibodies; matrix 1 (M1) proteins are targeted by T cells (80); and nucleoproteins are targeted by T cells (80) and nonneutralizing antibodies (81).

### Experimental Paradigms of Maternal Infection

While maternal infection is reported to be a risk factor for schizophrenia, controversy remains concerning which biological processes underlie this risk. There is scant evidence for transplacental passage and persistence of the influenza virus in the offspring brain (82). More likely to be relevant are the effects of infection-induced maternal immune activation (MIA) on the developing brain (83).

MIA cannot be easily modelled in humans and longitudinal, prospective research on effects of infection during pregnancy on human development is scarce (84). Hence, translational animal models of MIA have been developed: these models have been critical in providing causality to the epidemiological data and are starting to provide clues as to the cellular and molecular mechanisms that may underlie the associations (83). Rodents in gestational periods are exposed either directly to a pathogen such as influenza, or more commonly to nonvirulent immune-activating agents such as the viral mimetic polyriboinosinic-polyribocytidilic acid (poly(I:C)) or the bacterial endotoxin lipopolysaccharide (LPS), the inflammatory agent turpentine, or specific proinflammatory cytokines (83, 85). These animal models of MIA provide evidence for behavioral, neurochemical, neuroanatomic, and neurophysiologic disruptions in the offspring which map onto endophenotypes observed across human psychiatric disorders with a neurodevelopmental onset (83, 86). Such translational research complements the insights from human epidemiology by establishing causal relationships, identifying cellular and molecular mechanisms and offering the potential to explore therapeutic interventions (85, 86). Frequently, these aforementioned deficits in the MIA model demonstrate a maturational delay, such that they are not evident before young adulthood, and many studies have sought to mitigate these deficits with treatments (e.g., pharmacological, immunological, behavioral) (83). Another important etiological advance for such animal models is to recapitulate a “two-hit” approach, in which pathology becomes evident in MIA-exposed offspring only after a second hit, such as unpredictable psychological stress (87).

MIA may lead straightforwardly to damage to the foetal brain during the early stages of neurodevelopment (82), but may also provide entry into a deviant trajectory of neural development which predisposes offspring to behavioral deficits depending on the intensity of the infection and when in gestation it occurs [early vs. late—and potentially as late as the lactation stage (88)]. MIA-associated abnormalities have been described, sometimes inconsistently, for multiple brain cell types, all of which are implicated across psychiatric disorders from postmortem data and genetic studies to a greater or lesser extent: Schwann cells (89), astrocytes and microglia (90, 91), hippocampal GABAergic cells (92, 93), dopaminergic neurons (94), and parvalbumin interneurons (95, 96).

Notably, most rodent (and primate) MIA models use a dose of poly(I:C) which models a high intensity, acute and transient (<24 h) infection, the physiological relevance of which could be questioned. Furthermore, factors such as the source, molecular weight, and endotoxin contamination of experimental poly(I:C) may be unrecognised sources of variability in foetal outcomes (97). Although MIA models specifically using pathogens as the immune activating stimulus have become rarer in recent years, primarily due to increasingly stringent safety frameworks around the use of potentially virulent pathogens (98), a series of MIA studies using human H1N1 influenza infection by the group of S. Hossein Fatemi are particularly valuable in elucidating potential mechanisms of psychosis risk. Broadly, maternal human H1N1 infection has been demonstrated to cause abnormalities, within the offspring, of the following [summarized in (99) and (59)]:

*Gene Expression*: the breadth of gene expression changes was greater the later in embryonic development infection occurred; for example embryonic day 16 or 18 (E16 or E18) infection disrupted more genes, across more brain regions, than did E7 infection. Furthermore, infection at later embryonic stages disrupted expression of genes involved in myelination and implicated in schizophrenia risk.

*Protein Expression:* increase in production of potentially harmful neuronal nitric oxide synthase (nNOS), reduction of reelin expression indicating abnormal neuronal migration and decreased synaptic plasticity, and downregulation of myelin basic protein.

*Brain Structure:* reduced cerebral cortical volume; increased total brain volume after early embryonic infection, decreased total brain volume after late infection.

*Behavior:* decreased prepulse inhibition (PPI), increased head twitch response.

*Neurotransmitter Levels:* reduced serotonin and taurine levels.

*Placental Development:* increased cytoarchitectural disorganisation, increased presence of immune cells, presence of variously sized thrombi, and dysregulation of placental gene expression.

Additional selected studies, focused on models of infection with influenza virus, are presented in **Table 2**.

**Table 2:** Summary of selected behavioral and pathological outcomes following influenza infection in rodents.

Study	Year	Influenza virus type	Animal	Animal infected	Age of animal at assessment	Behavioral and physical outcomes	Pathological outcomes
Cotter et al. (100)	1995	A/Singapore/1/57 (H2N2)	Mice	Mice between day 9-16 of pregnancy	Offspring 21 days postpartum	N/A	No excess pyramidal cell disarray when compared with influenza-free, age-matched controls. Cell disarray greater among mice exposed on day 13 of pregnancy
Fatemi et al. (101)	1998	A/WSN/33 (H1N1)	Mice	Mice on day 9 of pregnancy	Neonate pups at postnatal day 0 (P0; day of delivery)	N/A	Increased expression of membrane protein synaptosome-associated protein 25 kDa [SNAP-25), a presynaptic neuronal marker in the neonatal brain: 40%–347% over control in most septal–dorsal hippocampal layers; 10%–114% over control in all mid septo-temporal hippocampus layers, except for the hippocampal plate; but SNAP-25 expression was reduced in all temporal–ventral levels, infected layers by 21%–33% below control except for mild increases of 8.8% and 10% in subplate and hippocampal plate layers
Fatemi et al. (102)	1999	A/WSN/33 (H1N1)	Mice	Mice on day 9 of pregnancy	Neonate pups at P0	N/A	Changes influencing levels of reelin, a protein responsible for normal lamination of the brain. Significant reductions in reelin-positive cell counts in layer I of neocortex and other cortical and hippocampal layers. Layer I Cajal–Retzius cells produced significantly less reelin. Decreases in neocortical and hippocampal thickness
Fatemi et al. (103)	2000	A/WSN/33 (H1N1)	Mice	Mice on day 9 of pregnancy	Adolescent offspring (P35) and young adults (P56)	N/A	Changes in the levels of neuronal nitric oxide synthase (nNOS) involved in synaptogenesis and excitotoxicity: increase of 147% in nNOS levels in the brain at P35, with an eventual 29% decrease on P56. Reductions in nNOS in middle and caudal brain areas on P35 and P56.
Aronsson et al. (104)	2001	A/WSN/33 (H1N1)	Mice	Four-week-old *Tap1* (antigen peptide transporter 1) gene knockout mice	7 days and 10, 12, and 17 months p.i.	N/A	Viral RNA encoding the nonstructural NS1 protein was detected in sections at midbrain levels in most animals. Negative-strand genomic RNA and positive-strand RNA, including mRNA, were found. RNA encoding nucleoprotein and polymerases, which form the replicative complex of the virus, were detected in fewer brains. RNA encoding envelope proteins were found only in occasional brains. No viral cDNA could be identified
Aronsson et al. (82)	2002	A/WSN/33 (H1N1)	Mice	Mice on day 14 of pregnancy	Foetuses at pregnancy day 17; offspring 10, 20, 35, 60, and 90 days of age	N/A	Viral RNA encoding matrix and/or nucleoprotein detected in a proportion of foetal brains and lungs, viral RNA detected in some placentas. RNA persisted for at least 90 days of postnatal life
Fatemi et al. (105)	2002	A/WSN/33 (H1N1)	Mice	Mice on day 9 of pregnancy	Offspring at P0, P14 and P35	N/A	Altered expression of glial fibrillary acidic protein (GFAP), a marker of gliosis, neuron migration, and reactive injury: increases in GFAP-positive density in exposed cortical and hippocampal cells; ependymal cell layer GFAP-IR cell counts showed increases with increasing brain age from P0 to P14 and P35 in infected groups. The GFAP-positive cells in showed ‘hypertrophy' and more stellate morphology
Fatemi et al. (106)	2002	A/WSN/33 (H1N1)	Mice	Mice on day 9 of pregnancy	Neonates at P0 and 14-week-old offspring	One exposed group with deficient prepulse inhibition (PPI), one group did not show abnormal PPI	The rate of pyramidal cell proliferation per unit area decreased from birth to adulthood in both control and exposed groups, nonpyramidal cell growth rate increased only in the exposed adult mice
Shi et al. (107)	2003	A/NWS/33CHINI (H1N1)	Mice	Mice on day 9.5 of pregnancy	Adult offspring	Deficient PPI; deficient responses to acute administration of clozapine, chlorpromazine and ketamine; deficient exploratory behavior in open-field and novel-object tests; deficient social interaction	N/A
Asp et al. (108)	2005	A/NWS/33 (H1N1)	Mice	Mice on day 14 of pregnancy	Offspring sampled at E17 and sex-matched animals on P35, P60, and P90	N/A	Levels of transcripts encoding neuroleukin and fibroblast growth factor 5 were significantly elevated in the brains of the virus-exposed offspring at 90 and 280 days of age, but not at earlier time-points. For neuroleukin, this difference could also be observed at the protein level
Fatemi et al. (109)	2005	A/NWS/33 (H1N1)	Mice	Mice on day 9 of pregnancy	Newborn offspring	N/A	Significant upregulation of 21 genes and downregulation of 18 genes in brains of day 0 exposed offspring, including genes involved in signal transduction/cell communication, solute transport, protein metabolism, energy metabolism, nucleic acid metabolism, immune response, and cell growth and maintenance
Asp et al. (110)	2007	A/NWS/33 (H1N1)	Mice	Newborn offspring infected on P3	Newborn offspring on P3; whole brains were sampled at: embryonal day (E)17 and P7, P13, and P24. From two animals, the hippocampus, cortex, and cerebellum were dissected from freshly prepared brains at P3, P10, P15, and P27	N/A	Increased levels of transcripts encoding Gcm1 and syncytin B, but not syncytin A, in NIH-3T3 cells as well as in mouse primary neurons or glia. Overexpression of human GCM1 in NIH-3T3 cells resulted in increased levels of transcripts encoding syncytin B but not syncytin A. Systemic administration of neurotropic influenza A virus resulted in a neuronal infection and increased levels of Gcm1-encoding transcripts in brains of young mice
Fatemi et al. (111)	2008	A/NWS/33 (H1N1)	Mice	Mice on day 18 of pregnancy	Male offspring tested at birth (P0), childhood (P14), adolescence (P35), and young adulthood (P56)	N/A	Altered gene expression of Sema3a, Trfr2 and Vldlr and altered protein levels of Foxp2. Embryonic day 18 mother infection led to significant gene alterations in frontal, hippocampal and cerebellar cortices of developing offspring. Significant atrophy in several brain areas and white matter thinning in corpus callosum. Altered levels of serotonin (P14, P35), 5-Hydroxyindoleacetic acid (P14) and taurine (P35)
Fatemi et al. (112)	2008	A/NWS/33 (H1N1)	Mice	Mice on day 9 of pregnancy	Offspring tested at birth (P0), childhood (P14), adolescence (P35), and young adulthood (P56)	N/A	Changes in mRNA and protein levels of nucleolin, aquaporin 4, and connexin 43 (markers involved in ribosomal RNA transcription, potentially viral replication, water transport, and changes in brains of subjects with autism): nucleolin mRNA and aquaporin 4 significantly decreased in neocortex at P0 and P35. Protein levels were significantly upregulated at P35 and P56 in neocortex and P56 in cerebellum Microcephalin mRNA was significantly decreased in neocortex at P56 and protein levels were significantly decreased at P56 in the cerebellum
Fatemi et al. (113)	2008	A/NWS/33 (H1N1)	Mice	Mice on day 16 of pregnancy	Offspring at P35 and P56	N/A	Twofold or greater upregulation of 103 genes and downregulation of 102 genes in cerebellum at P35. Twofold or greater upregulation of 27 genes and downregulation of 23 genes in the cerebellum at P56. Genes with their regulation disrupted are involved in cell growth and/or maintenance, channel proteins, membrane receptors, signalling, and transcription regulation, among other functions
Holtze et al. (114)	2008	A/NWS/33 (H1N1)	Mice	Mice infected at infected at P3 or P4	Whole brains from both sexes sampled at P7, P13, or P24	N/A	Altered levels of transcripts encoding several key enzymes of the kynurenine pathway observed in the brain on P7 and P13 but not on day P24. On P13, infiltrating T lymphocytes and increased levels of kynurenic acid in the brains of the infected animals
Winter et al. (115)	2008	A/NWS/33 (H1N1)	Mice	Mice on day 16 of pregnancy	Male offspring tested at P0, P14, P35, and P56	N/A	A significant decrease in serotonin levels in the cerebella of offspring of virally exposed mice at P14. No differences in dopamine levels between exposed and control mice. A significant decrease in dopamine at P14 and P56 compared to P0
Asp et al. (116)	2009	A/NWS/33 (H1N1)	Mice	Wild-type mice and *Tap1* gene knockout mice infected at P3 or P4	3–4-months-old male mice	Infected *Tap1* gene knockout mice, but not wild type mice, exhibited deficits in working memory, increased rearing activity, and anxiety	Reduced levels of type III *Nrg1* transcripts in the medial prefrontal cortices of *Tap1* gene knockout mice were observed. The lack of CD8^+^ T cells appeared to contribute to a more pronounced glia response in *Tap1* gene knockout mice than in wild-type mice
Shi et al. (117)	2009	A/NWS/33CHINI (H1N1)	Mice	Mice on day 9.5 of pregnancy	Adult offspring and offspring 11 days of age	N/A	Purkinje cells deficit in the cerebellum
Fatemi et al. (118)	2009	A/NWS/33 (H1N1)	Mice	Mice on day 16 of pregnancy	Male offspring tested at P0, P14, P35, and P56	N/A	Altered expression of myelination-related genes, including *Mbp*, *Mag*, and *Plp1*, and altered levels of proteins Mbp, Mag, and DM20. Significant atrophy in cerebellum at P14, reduced fractional anisotropy in white matter of the right internal capsule at P0, increased fractional anisotropy in white matter in corpus callosum at P14 and right middle cerebellar peduncle at P56
Fatemi et al. (119)	2009	A/NWS/33 (H1N1)	Mice	Mice on day 16 of pregnancy	Male offspring tested at P0, P14, P35, and P56	N/A	Altered gene expression in the hippocampus at P0, P14, and P56 including *Aqp4*, *Mbp*, *Nts*, *Foxp2*, *Nrcam*, and *Gabrg1*. Downregulation of myelination genes *Mag*, *Mog*, *Mobp*, *Mal*, and *Plp1* at P0. Reduction in hippocampal volume at P35
Asp et al. (120)	2010	A/WSN/33 (H1N1)	Mice	Wild-type mice and *Tap1* gene knockout mice infected at P3 or P4	Male mice at age 5–6 months tested for PPI; whole brains of the *Tap1* gene knockout mice sampled at P7, P13, and P24 to explore the kynurenine pathway	Tap1 gene knockout mice, but not wild-type mice, exhibited a reduction in PPI at 5–6 months of age	Levels of several transcripts in the kynurenine pathway altered at P7, P13 and P24. Transcripts encoding indoleamine-pyrrole 2,3-dioxygenase (IDO), degrading tryptophan in the first step of the kynurenine pathway were consistently up-regulated
Moreno et al. (121)	2011	A/WSN/33 (H1N1)	Mice	Mice on day 9.5 of pregnancy	Adult offspring (10–12 weeks of age)	Increased head-twitch response to hallucinogens, diminished antipsychotic-like effect of the glutamate agonist	In frontal cortex, the upregulated 5-HT(2A) receptor and the downregulated mGlu(2) receptor. The cortical 5-HT(2A) receptor-dependent signalling pathways altered, showing higher c-fos, egr-1, and egr-2 expression in response to the hallucinogenic drug DOI
Landreau et al. (94)	2012	A/New Caledonia/20/99-like (H1N1) (A/NC-L/99), A/Sydney/5/97-like (H3N2) (A/Sy-L/97), A/WSN/33 (H1N1)	Rats and mice	Primary cultures of rat mesencephalon infected after day 14 of pregnancy; mothers on day 9–11 of pregnancy	Neurons from rat embryos recovered at day 14 of pregnancy; offspring of mice infected in pregnancy tested at 30 and 90 days of age	The A/WSN/33 strain associated with greater behavioral impairment (exploration, novel objects, and spontaneous activity) than A/NC-L/99. Offspring of mother infected with both influenza virus strains showed behavioral abnormalities in exploration, anxiety and working memory. Behavioral alterations emerged in different neurodevelopmental stages depending on the strain, appearing in adult life in offspring of mothers infected with A/NC-L/99	Selective loss of dopaminergic neurons. H1N1 strains had the greatest affinity for dopaminergic neurons, an H3N2 strain induced apoptosis preferentially in other cell types and did not result in NFkB activation. Only following the H1N1 strains infection a selective loss of dopaminergic neurons in substantia nigra pars compacta and ventral tegmental area of the offspring. Loss of dopaminergic neurons more pronounced in the adult offspring of mothers infected with the neuroadapted A/WSN/33 than with the respiratory strain A/NC-L/99
Fatemi et al. (122)	2012	A/WSN/33 (H1N1)	Mice	Mice on day 7 of pregnancy	Placentae of pregnant mice; male offspring tested at P0, P14, P35, and P56	N/A	Upregulation of 77 genes and significant downregulation of 93 genes in placentas. Changes in gene expression in prefrontal cortex (6 upregulated and 24 downregulated at P0; 5 upregulated and 14 downregulated at P56) and hippocampus (4 upregulated and 6 downregulated at P0; 6 upregulated and 13 downregulated at P56) of exposed offspring. Placentas from infected mice with morphological abnormalities including presence of thrombi and increased presence of immune cells. No H1N1 viral-specific genes for M1/M2, NA, and NS1 in placentas of infected mice and brains of exposed offspring
Fatemi et al. (123)	2017	A/NWS/33 (H1N1)	Mice	Mice on day 16 of pregnancy	Male offspring tested at P0, P14, P35, and P56	N/A	Changes in proteins FMRP, VLDLR, GAD65, and GAD67 in cerebella of exposed offspring on specific postnatal dates which implies disrupted FMRP, glutamatergic, and Reelin signalling leading to developmental abnormalities

Other animal studies have demonstrated associations between maternal influenza infection and schizophrenia-related neurotransmitter dysfunction including elevated serotonin 5-HT2A receptor expression in the frontal cortex (121), reductions of cerebellar serotonin levels at postpartum days (P) 14 and P35 (111, 115), downregulation of the metabotropic glutamate receptor 2 in the frontal cortex (121), and decrease in dopamine levels at P14 and P56 (115). Changes following poly(I:C) MIA exposure included subtle metabolic perturbations of postnatal prefrontal cortex maturation (124), and dynamic changes in volumes of multiple brain structures (125), including adult changes which can be prevented by periadolescent administration of antipsychotic medication (at nonantipsychotic dose equivalents) (126). Supporting the translational relevance of these studies, there is an emerging parallel literature in humans suggesting that early immune activation affects subsequent brain development and behavior: for example, maternal IL-6 levels during pregnancy predicted greater neonatal amygdala volumes and connectivity, which in turn predicted poorer impulse control at two years of age (127); complementary results for amygdala connectivity and internalizing behaviors have been reported for maternal cortisol levels (128).

An important mediator of the maternal immune response to infection is likely to be disruption of cytokines regulating brain development. Notably maternal infection could dysregulate cytokine networks either by direct transplacental transfer of cytokines to the foetus, by placental cytokine production or by increased foetal production of cytokines, including within the CNS (129). Cytokine dysregulation can result in perturbations of both proinflammatory and antiinflammatory cytokines. The deleterious or protective effects of any individual cytokine are likely determined by its context within a network of proinflammatory and antiinflammatory mediators, dynamically responding to external and endogenous challenges with differential expression in different brain regions over time (130). For example, macrophage-driven expression of antiinflammatory IL-10 in a mouse model can attenuate the long-term effects of prenatal viral infection, but in the absence of inflammatory stimulus, IL-10 itself precipitates offspring behavioral abnormalities (131).

Cytokines are induced in response to inflammation by neurons, astrocytes, and microglia, with the role of activated microglia in schizophrenia pathogenesis being the object of much recent attention (132). MIA exposure leads to alterations of the microglial transcriptome, with an initial shift to a more reactive state proximal to the MIA insult, followed by a delay in the maturation of brain microglia, as compared to controls (133). Alterations in the microglial transcriptome also lead to phagocytic function abnormalities and behavioral abnormalities in the adult MIA offspring (134). Results from the MIA literature are heterogenous with regards to microglial activation in offspring (91). Some studies report increased microglial density [e.g. (135)], morphology [e.g. (136)] or expression of activation markers [e.g. (137)], while other studies using late MIA have failed to demonstrate long-term changes in microglia density, morphology, or activation (138). Inference regarding the translational relevance of these findings, too, has been limited by a lack of clarity around the utility of the putative human markers of microglial activation such as translocator protein (TSPO), bound by ligands in Positron Emission Tomography (PET) studies, and of microglial markers used in post-mortem studies; notably, in addition to microglia, both astrocytes and vascular endothelial cells show dynamic changes in TSPO expression in response to inflammatory stimuli, and in a mouse model schizophrenia-relevant behavioral abnormalities and increased inflammatory cytokine expression were associated with reduced, rather than increased, prefrontal TSPO levels (139).

Both neurotropic and nonneurotropic influenza strains can cause microglial activation and potentially contribute to inflammation (76, 77). Innate immune training against influenza confers protection against infection with antiviral interferon-stimulated defence genes, including *MXA* (prevents nuclear import of the virus), *IFITM3* and other *IFITM* proteins (block host-virus cell membrane fusion), and viperin [blocks influenza virus release; (140)]. Innate immune training also promotes disease tolerance of host tissues (140) and previous activation primes microglia to respond strongly to a new stimulus (141). Previous neuropathology potentially attunes microglia to respond more strongly to systemic inflammation (142), including inflammation by chronic mild stress in periadolesence following MIA (86). Consequently, infection could prime microglia towards heightened activation, potentially increasing the risk of developing psychotic symptoms (143); alternatively, the opposite could be true, i.e., that the microglia become tolerant and as such cannot respond flexibly to new stimuli.

In terms of potential downstream consequences of such foetal microglial activation, there is an emerging literature implicating the role of microglia in shaping brain development, including the potential for synaptic pruning *via* the full or partial engulfment [phago- or trogocytosis (144)] and putative degradation of synaptic inputs, a process mediated at least in the rodent visual thalamus in a complement and activity-dependent manner (145), which may also involve other molecular mediators such as TREM2 for example or the fractalkine receptor [CX3CR1; (146)] [potential mechanisms are reviewed in (147)]. Given the well-replicated finding of reduced dendritic spine number in schizophrenia (148), and evidence that patient-derived microglia-like cells are capable of synapse elimination at least *in vitro* (149), it is plausible also that maternal infection-induced foetal microglial activation could lead to later psychopathology through upregulation of synaptic pruning mechanisms (or conversely, loss of them: for example, early in development, microglia contact of neurons actually stimulates dendritic spine formation (150) suggesting the underexplored possibility that different pathologies play out in a potentially time- and region-specific manner).

Additionally, influenza infection may lead to placental abnormalities that result in hypoxia and/or nutritional deficiency or foetal brain growth restriction (122). Brain abnormalities may also stem from the maternal immune response whereby maternal autoantibodies are transported *via* the placenta and interact with foetal brain antigens to disrupt brain development [the teratogenic antibody hypothesis of schizophrenia; (151–153)]. The concept of infection-induced brain autoimmunity is explored in the next section.

## Influenza and Autoimmunity

Research on autoimmune disorders and schizophrenia dates to the 1950s, when schizophrenia was found to be protective against the development of rheumatoid arthritis (154, 155). Subsequently, the cooccurrence of schizophrenia with celiac disease was noted [e.g. (156)]. Recent meta-analysis suggests a positive association between nonneurological autoimmune disorders and psychosis (157). The risk for developing schizophrenia in people with autoimmune disorders was found to increase in association with increasing number of hospitalisations for infections (158), suggesting a synergistic effect. Drawing on the clinical observation that patients with autoantibody-mediated encephalitis frequently presented with psychosis (159), serological research has reported the presence of these same autoantibodies against cell surface neuronal antigens in some patients with schizophrenia [e.g. (160, 161)]. An extensive literature also focuses on markers of previous infection (usually IgG antibodies to specific pathogens, including influenza) in adults with psychotic disorders, with exposure to several organisms associated with increased schizophrenia risk or risk for a specific psychosis symptom profile (e.g. impaired cognition) (17, 162).

### Viral infection, Neuronal Surface Autoantibodies and Psychosis: Anti-NMDAR Encephalitis as a Model of Autoimmune Psychosis With Potential Infective Antecedents

Autoimmune encephalitis (AE) frequently presents with acute psychosis in adults (163, 164). Autoantibodies to a variety of CNS cell surface antigens (neuronal surface autoantibodies; NSAbs) have been implicated in AE, including the NMDAR and more rarely LGI1, CASPR2, AMPAR, GABA_A_R, GABA_B_R, D2R, DPPX, mGluR5, and GlyR (165–169). Of the autoimmune encephalitides associated with the above antigens, NMDAR encephalitis presents most frequently with psychosis. The typical pattern includes prodromal malaise, or influenza-like symptoms, before the emergence of psychiatric symptoms. 4% of patients show isolated psychotic episodes at presentation or relapse (170) and behavioral and cognitive impairments including psychosis are predominant early symptoms (171). The psychosis reported in anti-NMDAR encephalitis is distinctive, polymorphic (with significant affective elements) and does not correspond clearly to currently existing categories of psychotic disorder in mainstream psychiatric use (172). Anti-NMDAR encephalitis is caused by IgG antibodies directed against an epitope on the N-terminal domain of the NR1 subunit of the NMDA glutamate receptor (173, 174), with intrathecal antibody production by B lymphocyte descendants thought to be essential for pathogenesis.

Anti-NMDAR encephalitis is associated with ovarian teratoma in under a third of cases. An intriguing association between infection and the development of the disorder became apparent when it was observed that a number of patients experiencing “relapses” following herpes simplex virus (HSV) encephalitis had cerebrospinal fluid (CSF) NMDAR antibodies, suggesting that these “relapses” were in fact a postinfectious AE, rather than the result of reinfection or viral reactivation (175). Subsequent work has established that NMDAR antibody production can occur following HSV encephalitis even in the absence of clear “relapse” or encephalopathy (176), and that nonencephalitic HSV infection is also more common in patients with anti-NMDAR encephalitis (177). Other viral pathogens—including Epstein Barr Virus, Human Herpesvirus 6, cytomegalovirus, adenovirus and HIV—have been implicated in this and other autoimmune encephalitides [including those characterized by antibodies to the GABA_A_ and GABA_B_ receptors, the AMPA receptor and the dopamine D2 receptor; reviewed in (178)].

A potential association between anti-NMDAR encephalitis and influenza is supported by reports of at least five patients who developed the disorder following influenza vaccination (179–182)—although in none of these cases can causation be proven. A phylogenetic relationship has been suggested between microRNAs related to anti-NMDAR encephalitis and the H1N1 influenza virus, with some authors suggesting a theoretical basis for the possibility that anti-NMDAR encephalitis could be induced by influenza vaccination [(183); see also next section].

Anti-NMDAR encephalitis shares clinical features with EL in children, and indeed NMDAR antibodies have been reported in children with contemporary EL (184). When considered in the light of classic research on EL following the 1918-1919 Spanish influenza pandemic (32), this suggests a potential relationship between influenza infection, NSAbs and psychosis in anti-NMDAR encephalitis. [The association between influenza and EL is, however, highly controversial, not least because of temporal and geographical discrepancies between the start of the pandemic and the first recorded EL cases, as well as studies on post-mortem tissue which have frequently failed to find evidence of influenza virus; but given historical issues with case ascertainment and storage of biological samples potentially undermining efforts at viral detection, the association remains a plausible hypothesis for some authors (185)].

This interpretation is supported by findings that anti-NMDAR encephalitis may be seasonal, with a peak in incidence during winter (186), potentially converging with seasonality of influenza. Recent research found that Māori and Pacific Island populations have higher incidence and potentially more severe outcomes of anti-NMDAR encephalitis, a finding of significance given that population's apparent increased susceptibility to severe influenza infection (187, 188).

NMDAR antibodies are of interest in schizophrenia because of their links to the glutamate/NMDAR hypofunction hypothesis of psychotic disorders: NMDAR antibodies found in patients with schizophrenia can disrupt NMDAR dynamics *in vivo* (189, 190) providing *prima facie* support for the NMDAR hypofunction hypothesis. Crucially, Hammer et al. (191) reported the presence of influenza virus A or B IgG was significantly associated with NMDAR antibody seropositivity in a large cohort of adult patients with psychotic disorders and disease and healthy controls, a finding that was subsequently replicated in an independent cohort (192).

### Acquired Neuronal Autoimmunity and Its Relevance to the Maternal Exposure Model

As described above, infection-induced neuronal autoimmunity may have relevance for some acute psychoses. However neuronal autoimmunity also has relevance in the context of maternal transmission. Maternal-foetal transfer of pathogenic antibodies has long been proposed as a potential mechanism in the development of ASD and, to a lesser extent, for schizophrenia also (153). Although not formally regarded as part of the MIA paradigm, recent animal models have had some successes in recapitulating neurodevelopmental phenotypes in immunisation paradigms whereby maternal antibodies are transferred to the offspring, resulting in neuropathological and behavioral abnormalities (193–195). Two of these studies used CASPR2 antibodies, cell surface IgG antibodies which have been implicated in encephalitis and a variety of peripheral nerve manifestations. Intriguingly, a study by Coutinho et al. found that NMDAR antibodies were more frequent in mothers of children with neurodevelopmental disorders, who themselves (i.e. the mothers) subsequently developed psychosis. This finding was not replicated in another cohort in which the mothers did not go on to develop psychosis, but clearly mandates attempts at replication (196). A recent animal study has shown that maternal-foetal transfer of recombinant NMDAR NR1 antibodies—at levels that did not affect the behavior of the pregnant mother—resulted in impaired neurodevelopmental reflexes, reduced anxiety, motor hyperactivity, and impaired sensorimotor gating, the latter two of which were regarded as psychosis-like phenotypes (197) (but see section “*Experimental paradigms of maternal infection*” for transdiagnostic relevance of these behaviors).

### Influenza and Molecular Mimicry

The association between influenza infection and NMDAR autoantibody status may have structural molecular basis. The influenza A M2 channel and NMDAR share a ligand, the antiviral compound amantadine (198), suggesting putative structural homology which could form the basis for NMDAR autoimmunity occurring after infection. In molecular mimicry, there is sharing of sequences, such as linear amino acid sequences, by molecules from dissimilar genes or their protein products. In infection, if the virus shares cross-reactive epitopes for B or T cells with the host, the host immune cells can target both the infecting agent and the host itself, potentially inducing autoimmune disease (199). The processes involved include T_c_ cells damaging self-tissue by lysis or T_h_ cells releasing cytokines. Cytokines in turn activate macrophages or stimulate secretion of antibodies, and antibodies bind to cross-reactive epitopes on the surface of tissues, triggering further cytokine production by macrophages (200). Damaged tissues can also release new self-epitopes which activate autoantigen-reactive T and B cells, recognising those self-epitopes [epitope spreading; (201)].

There are multiple strands of evidence that influenza infection may have an aetiological role in systemic autoimmunity, including in Henoch-Schonlein purpura, type 1 diabetes mellitus and antiphospholipid syndrome [reviewed in (202)]. In one study, influenza vaccination induced autoimmunity (primarily antiphospholipid antibodies) in apparently healthy volunteers (203). H1N1 infection in rabbits has also been shown to induce brain-reactive antibodies, including to a 37kDa target also present in humans (204). Precedent for the role of influenza exposure initiating neurological disorder, potentially *via* molecular mimicry, exists for Guillain-Barre syndrome (205) and narcolepsy, in which hypocretin-producing neurons could be an autoimmune target due to molecular mimicry between H1N1 virus-derived antigen and a neuronal autoantigen in HLA-DQB1*06:02 positive patients (206, 207); see (208) for an example model of narcolepsy. This association is supported by epidemiological findings of an increased risk of narcolepsy in children following the H1N1 vaccination, Pandemrix (209, 210), and by serological findings that antibodies to influenza nucleoprotein might cross-react with hypocretin receptor 2 in patients with Pandemrix vaccination [(211), although see (212–214)].

Further evidence for molecular mimicry as a bridging link between influenza infection, the adaptive immune response and neurodevelopmental risk for schizophrenia comes from gene sequence overlap between the H5N1 virus and genes abnormally regulated in schizophrenia (215). Furthermore, the H1N1 influenza antiviral protein hemagglutinin was found to share peptide structure with a variety of human axon guidance proteins; the majority of proteins identified as containing homologous sequences are involved in processes which, if disrupted, could lead to deviant neurodevelopmental trajectories. The observed peptide matches were conserved across influenza strains and frequently involved experimentally validated hemagglutinin epitopes (216). Finally, the NMDAR 2A subunit was found to share peptides with several pathogens, including the influenza A virus (217). The findings suggest that anti-pathogen immune responses to the influenza A virus may cross-react with multiple schizophrenia-related proteins. This reaction could potentially trigger processes which may ultimately lead to schizophrenia. Work from our group has confirmed the higher-than-expected overlap between the influenza proteome and schizophrenia-relevant proteins, additionally identifying hemagglutinin as contributing, amongst influenza proteins, the most extensive peptide sharing [Kepinska et al., *in submission*; see also (218)].

## Conclusion and Future Directions

**Figure 2:**
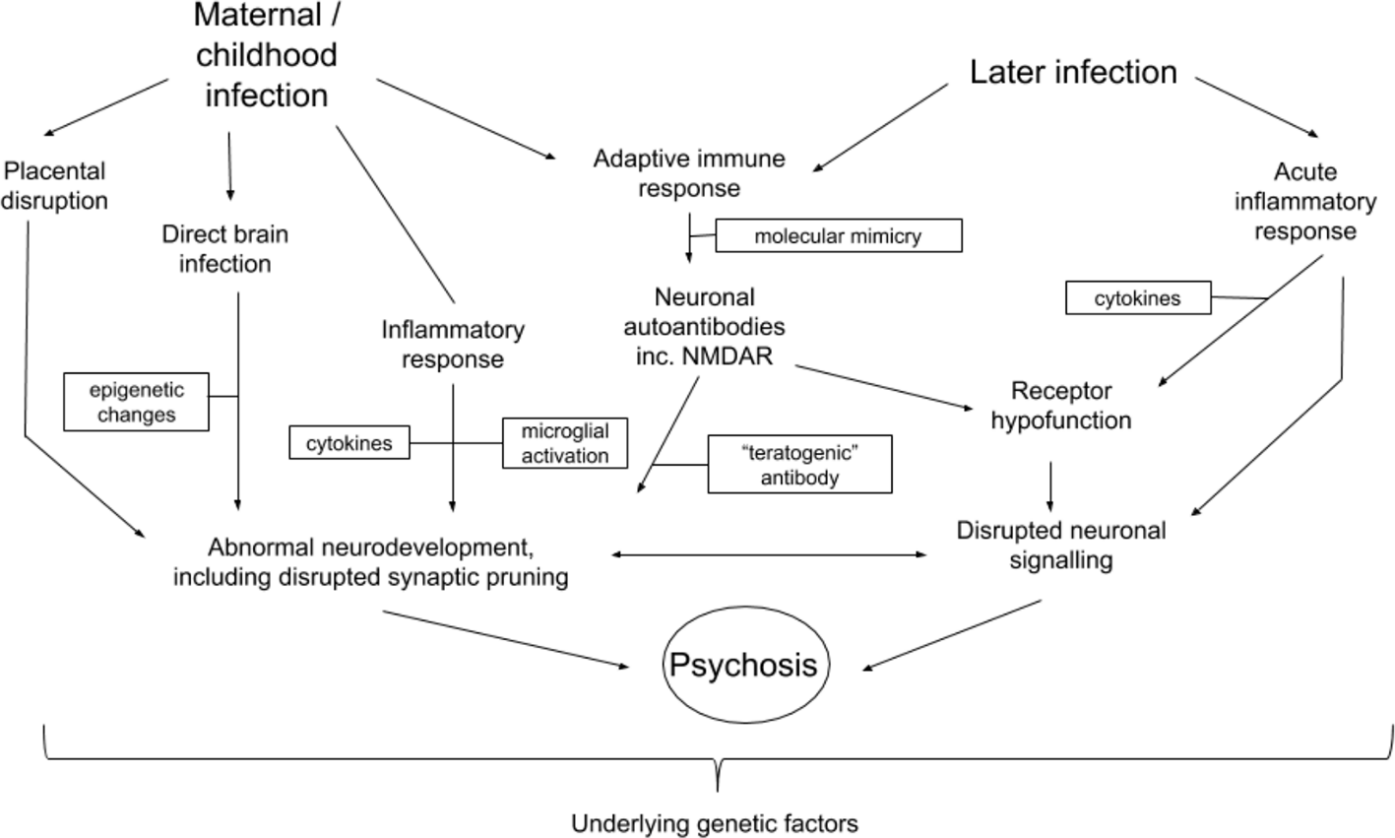
Potential interactions between mechanisms related to influenza infection and development of schizophrenia or other psychotic disorders. Arrows indicate possible directions of interaction. Boxes represent different factors or changes which mediate processes possibly leading to the development of psychosis or schizophrenia.

Converging evidence demonstrates that infection with the influenza virus has a multiplicity of effects on prenatal and postnatal processes which, when disrupted, could result in increased risk of the development of schizophrenia or acute psychoses in adulthood. **Figure 2** outlines potential prenatal and postnatal pathogenic contributions. Nonetheless, it is important to emphasise that infection has been linked with increased risk of several psychiatric disorders (see *Introduction*). It is therefore not clear to what extent the mechanisms discussed in this review are schizophrenia-specific, or whether, as is highly likely, other factors may shape the clinical expression of disease.

Outstanding questions and possible future experimental approaches are summarized in **Box A**.

**Box A | Outstanding questions for future research.**

- Do influenza MIA models, or models of adulthood influenza infection, demonstrate an antibody response to brain antigens including, for example, the NMDAR?
- Is there an epidemiological association between influenza vaccination (maternal, childhood or adulthood; seasonal or pandemic) with differential risk of subsequent development of psychotic disorder?
- Is antiviral use in pregnancy associated with a reduced risk of psychotic disorders in offspring?
- Is the acute response to influenza infection or vaccination in healthy individuals instructive for understanding the development of psychosis? Relevant approaches could include neuroimaging and behavioral testing following vaccination, similar to paradigms using LPS or typhoid vaccine administration (219–221).
- Will next generation viral metagenomic sequencing reveal differential presence of virus in biofluids from patients with psychotic disorders?
- Can an *in silico* approach be used to assess the plausibility of the molecular mimicry hypothesis, potentially assessing linear or structural overlap between viral proteome and schizophrenia-relevant proteins?

Future immunity-focused research on schizophrenia and influenza should further explore the relationship between infection and the innate and adaptive immune response in schizophrenia using animal models and large-scale serological studies in patients at different stages of disease. To date, MIA models typically include very little deep immunophenotyping, and discussion of the adaptive immune response in these models has been almost entirely lacking. Standardised and more sensitive testing technologies are required, including improved noninvasive methods to assess central neuroinflammation in humans and nonhuman animals (222, 223).

Recent developments in stem cell technology suggest the possibility of using induced pluripotent stem-cell (iPSC) microglia-like cells [as per (149)] to assess how influenza infection affects the phenotype of these cells. Potentially, iPSC-derived cerebral organoids [so-called ‘mini brains’ (224)] could offer a window into the effects of influenza infection on relevant aspects of neurodevelopment.

While this paper reviews limited case studies and series indicating that in some instances influenza vaccination has been linked to CNS-directed autoimmunity, there is currently no evidence demonstrating a clear association between influenza vaccination and the development of schizophrenia or other psychotic disorders. The limited reported cases constitute a weight of evidence which is far weaker than the many epidemiological studies supporting the association between maternal influenza infection and schizophrenia. Influenza vaccination—both pandemic and seasonal—has saved and continues to save countless millions of lives worldwide, with an overwhelming evidence base supporting its efficacy. Within this context, influenza vaccination may nonetheless represent an as-yet underutilised opportunity for epidemiological and mechanistic explorations of potential influenza-psychosis associations. For example, healthy volunteers having the vaccination could be assessed using immunophenotyping, brain imaging, and behavioral measures to further characterize the acute response to influenza exposure [analogous to similar human studies of the acute response to LPS or typhoid vaccine administration (219–221)].

From the perspective of prevention of psychosis, consideration has been given to the potential use of antiviral medication in at-risk pregnant women. Although human studies are lacking, pilot studies in mice suggest that giving oseltamivir to pregnant mice can prevent some influenza-induced changes in the offspring (99). And while oseltamivir is regarded as having a favourable profile in pregnancy, there are no data on the long-term effects on neurodevelopment in human children.

Consideration has also been given to the potential role of influenza vaccines prior to, or during, pregnancy as a preventive measure to limit the prenatal teratogenic influence of viruses (225–227). The seasonal influenza vaccine has established efficacy in preventing maternal infection, as well as partially preventing the infant through passive immunity, and its administration remains best practice for protection of mother and child, with the World Health Organisation recognising pregnant women as a priority vulnerable group. In addition, educating pregnant women to contact their healthcare provide if they have a fever is recommended in order to expedite administration of antiviral medication and supportive care (228). Some authors consider the fact that influenza vaccination is not recommended in the first trimester in some countries as cause for concern, leaving women and the developing foetus vulnerable during a critical neurodevelopmental window (228). Epidemiologically, first trimester (or any other trimester) pandemic influenza vaccination does not appear to be associated with increased childhood morbidity (229). Although neurodevelopmental outcome data are largely lacking, some mouse models suggested that influenza vaccination early in pregnancy can indeed promote behavioral function and neurogenesis in the offspring, and confer protection from the effects of MIA with LPS (230, 231). One note of caution has been raised by a cohort study of nearly 200,000 children in California which reported a small but statistically significantly increased risk of ASD following first trimester vaccination (232); unsurprisingly the report was controversial, with ensuing disagreement concerning interpretation of the findings and whether the correct statistical measures were used (233, 234).

Given that the vast majority of children of mothers who experience an infection do not develop psychiatric disease, recent consideration has been given to maternal and foetal mechanisms of resilience to perinatal infection and inflammation: these include maternal nutritional status, the microbiome, and a variety of postnatal environmental factors (235). In terms of interventions within the MIA paradigm that have potential widespread relevance, dietary supplementation with omega-3 polyunsaturated fatty acids (PUFAs) may represent an attractive preventative strategy (236, 237).

An increase in our understanding of neuro-immune interactions has enabled a fuller understanding of the mechanistic underpinnings of the neurodevelopmental hypothesis of schizophrenia and have contributed to a more nuanced picture of schizophrenia pathogenesis which can accommodate the influence of influenza infections after the perinatal period. Our understanding of both influenza and schizophrenia has changed immensely since the 1918-1919 pandemic. The development of next-generation genetic, immunological and bioinformatic technologies may bring a resolution of the centuries-old puzzle of the relationship between influenza and psychosis.

## Author Contributions

TP, RM, CI and AK developed the initial idea for the review. AK and TP wrote the first draft of the manuscript. AK, CI, AV, RY, RM and TP contributed to revision and editing of the manuscript.

## Funding

TP was supported in a Clinical Research Training Fellowship by the Wellcome Trust (grant number: 105758/Z/14/Z) and in a clinical lectureship by the National Institute for Health Research (NIHR). AK is funded by the NIHR Maudsley Biomedical Research Centre at South London and Maudsley Foundation Trust and King's College London. The views expressed are those of the author(s) and not necessarily those of the NIHR or the Department of Health and Social Care. AV acknowledges financial support from the Medical Research Council (New Investigator Research Grant (NIRG), MR/N025377/1). The work (at King’s College, London) was also supported by the Medical Research Council (MRC) Centre grant (MR/N026063/1). AV has received research funding from F. Hoffman La Roche Ltd and UBC Biopharma SPRL as part of a research programme on early life immune activation. The views expressed are those of the authors and not necessarily those of F. Hoffman La Roche or UCB Biopharma SPRL. These funders had no influence on the decision to publish this work.

## Conflict of Interest

The authors declare that the research was conducted in the absence of any commercial or financial relationships that could be construed as a potential conflict of interest.

The handling editor declared a past collaboration with the authors TP and RY.
